# Local food web management increases resilience and buffers against global change effects on freshwaters

**DOI:** 10.1038/srep29542

**Published:** 2016-07-08

**Authors:** Pablo Urrutia-Cordero, Mattias K. Ekvall, Lars-Anders Hansson

**Affiliations:** 1 Department of Biology/Aquatic Ecology, Lund University, Ecology building, SE-223 62 Lund, Sweden; 2 Center for Environmental and Climate Research, Lund University, Ecology Building, SE-223 62, Lund, Sweden

## Abstract

A major challenge for ecological research is to identify ways to improve resilience to climate-induced changes in order to secure the ecosystem functions of natural systems, as well as ecosystem services for human welfare. With respect to aquatic ecosystems, interactions between climate warming and the elevated runoff of humic substances (brownification) may strongly affect ecosystem functions and services. However, we hitherto lack the adaptive management tools needed to counteract such global-scale effects on freshwater ecosystems. Here we show, both experimentally and using monitoring data, that predicted climatic warming and brownification will reduce freshwater quality by exacerbating cyanobacterial growth and toxin levels. Furthermore, in a model based on long-term data from a natural system, we demonstrate that food web management has the potential to increase the resilience of freshwater systems against the growth of harmful cyanobacteria, and thereby that local efforts offer an opportunity to secure our water resources against some of the negative impacts of climate warming and brownification. This allows for novel policy action at a local scale to counteract effects of global-scale environmental change, thereby providing a buffer period and a safer operating space until climate mitigation strategies are effectively established.

Human-induced eutrophication has been one of the main environmental problems facing freshwater ecosystems during the past decades. The intensification of nutrient loadings (phosphorous and nitrogen) often leads to abrupt regime shifts in freshwater ecosystems, i.e., systems change from diverse, clear water states to turbid conditions with frequent occurrence of toxic phytoplankton (cyanobacteria) blooms^1^,^2^. Accordingly, eutrophic water bodies generally exhibit high abundances of zooplanktivorous fish, thereby eliminating efficient large-bodied grazers, such as the crustacean *Daphnia*^1^,^2^, and leading to massive algal growth, or so-called “blooms”. The effects of eutrophication are expected to intensify under climatic warming by further promoting cyanobacterial growth, which affect important ecosystem services such as the provision of drinking water and opportunities for recreation^3^,^4^,^5^. Hence, global-scale alterations in climate are likely to interact with local pressures on freshwaters, such as elevated nutrient levels and fish predation on zooplankton, and consequently affect ecosystem functioning and services^6^.

In addition, other environmental changes that act beyond the local scale are expected to occur simultaneously with global warming during the next decades. For example, the amounts of humic substances in inland waters have recently dramatically increased in northern temperate and boreal regions^7^,^8^. The cause behind this “brownification” seems to be a combination of several drivers, including reversal from acidification, warming and anthropogenic-driven changes in land-use and hydrological regimes^7^,^8^,^9^,^10^,^11^. Moreover, precipitation is expected to intensify in northern temperate regions in the future, thus increasing the runoff of humic substances and nutrients into water bodies^11^,^12^. The potential for interactive effects (e.g., synergistic effects) between these different environmental perturbations demands effective adaptive management tools if we are to secure the provision of freshwater for human societies and the functions of ecosystems^6^,^13^,^14^. Current experimental data^15^,^16^ show that global change effects are linked to the food web structure of ecosystems. However, to our knowledge, no studies to date have demonstrated this in natural systems by managing the food web structure to increase resilience against negative effects of global environmental changes.

Here we provide experimental evidence that the synergistic interaction between global climate warming and brownification of freshwaters will strongly stimulate cyanobacterial biomass in eutrophic systems. This increase in biomass is likely to elevate the amount of cyanobacterial toxins in freshwaters, thereby jeopardizing the future provision of safe water resources. However, we also demonstrate that manipulating the food web structure has the potential to reduce the mass proliferation of toxic cyanobacteria, showing that local-scale management actions may provide a temporal buffering against the effects of global environmental change.

Cyanobacteria show stronger responses to elevated temperatures than most phytoplankton^17^,^18^ and a warmer climate could therefore directly provide cyanobacteria with optimal environmental conditions for their growth. Furthermore, warm temperatures can indirectly enhance water column stratification and increase the sinking of phytoplankton competitors, which favours buoyant cyanobacteria owing to the production of intracellular gas vesicles^19^. Water column stratification can also create hypoxic conditions at the sediment surface, which subsequently benefits cyanobacteria by enhancing the internal loading of nutrients^2^. Brownification may also affect phytoplankton (and hence cyanobacteria) either positively or negatively via complex, interacting effects with the physical, chemical and biological environment^8^. One of the most severe effects is the increase in light attenuation by elevated humic content from the absorption of solar radiation^20^. Since cyanobacteria generally cope better with low light intensities than most phytoplankton^18^, increased brownification may amplify the positive effects of warming on cyanobacterial growth by relieving them from competition, provided a critical inhibiting threshold for photosynthetic activity is not surpassed^8^,^21^.

Here we use an experimental and field-based approach to test for potential interactions between temperature and brownification that might further affect freshwater quality, as characterized by the development of cyanobacteria and the presence of their toxins. Of specific interest was the response of the widespread cyanobacterium *Microcystis*, known to produce potent toxins to both humans and other animals^3^. The hepatotoxic microcystins, a family of over 80 variants of cyclic heptapeptides, are among the most common toxins produced by *Microcystis*^3^.

Two experiments were conducted in outdoor mesocosms that mimicked shallow water bodies. In order to separate the effects of climate and brownification, we used a factorial experiment with four treatments, hereafter referred to as “the crossing experiment”. The treatments included the present environmental conditions, an elevation in temperature based on the predictions from Intergovernmental Panel on Climate Change^12^, an increase in humic content based on trends observed in natural freshwaters^16^ and, finally, a treatment based on a future scenario that incorporated the predicted combination of climate warming and humic substances. To assess how different levels of the combined variables of climate and brownification are likely to affect water quality, we designed a complementary experiment hereafter referred to as “the gradient experiment.” In this experiment, we created a scenario of future changes aimed at forecasting the effects of predicted increases in temperature and humic substances in a time series. In order to relate the experiments to natural systems, we also assessed long-term monitoring data of the interannual variations in plankton, temperature and humic content (brownification) of the shallow and eutrophic Lake Ringsjön (southern Sweden), which has been subject to adaptive management actions (biomanipulation) since 2005 through the removal of cyprinid fish (Supplementary Fig. S1).

We predicted that elevated temperatures would favour the growth of toxic cyanobacteria over other phytoplankton taxa, mainly due to providing the optimum conditions for their growth^18^. Our studies focused on shallow lake ecosystems, which are common systems in peri-urban and agricultural areas, and therefore potential temperature-driven indirect effects on cyanobacterial growth from enhanced water column stratification are less intense compared to deep lakes^1^. In addition, we expected brownification to strengthen the positive relationship between temperature and cyanobacterial growth, given the low-to-intermediate level of humic content in our study systems (Supplementary Table S1). We also predicted that removing planktivorous and benthic-feeding fish through biomanipulation in Lake Ringsjön would increase the abundance of large, herbivorous zooplankton and reduce the rate of phosphorous release from sediments, making nutrients less available for algal growth in the water column^2^,^22^,^23^. Hence, we predicted that local food web effects created by this biomanipulation would counteract the negative effects of increasing temperature and brownification and thereby restrict cyanobacterial growth by altering both top-down (zooplankton grazing) and bottom-up processes (nutrient availability).

## Resuls and Discussion

**Figure 1:**
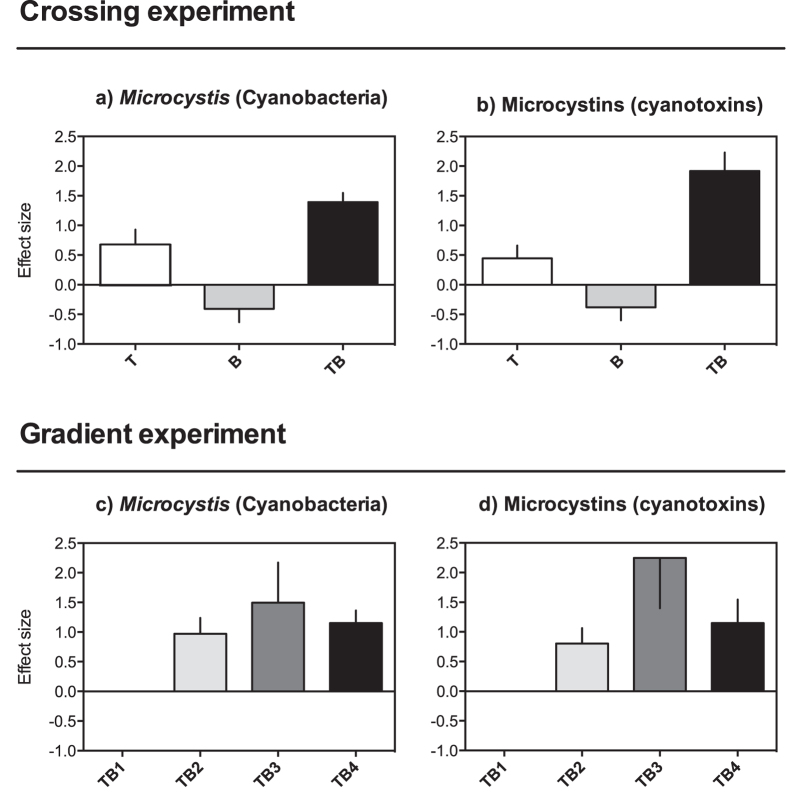
The effects of temperature and brownification on *Microcystis* (Cyanobacteria) and their toxins. The temporal mean effect sizes (±SE) of *Microcystis* and the microcystin concentrations in the crossing (**a**,**b**) and gradient (**c**,**d**) experiments. In (**a,b**): T is elevated temperature (+3 °C); B is brownification (double absorbance at 420 nm (used as a proxy for brownification)); and TB is the combination of both factors. In (**c,d**): TB1 is +1 °C and +50% absorbance; TB2 is +2 °C and +100% absorbance; TB3 is +3 °C and +150% absorbance; and TB4 is +4 °C and +200% absorbance. Note that the X-axis equals the control treatment and that values above or below that line indicate positive or negative treatment effects (warming and/or brownification), respectively, with respect to the control conditions^43^.

Our experiments show increasing biomass of cyanobacteria and toxin levels as a result of synergies between temperature and brownification, which supports the suggestion that the quality of freshwater will be compromised further during the lifetime of the next generation^6^,^12^. In both the crossing and gradient experiments, total phosphorous concentrations and phytoplankton biomasses (chlorophyll *a*) were not affected by any treatment (Supplementary Table S2). *Microcystis* biomass (μg L^−1^) and microcystin concentrations (ppb) seasonal average values (and ranges) across all treatments in both experiments were: crossing experiment: 877 (214–1632) and 1.92 (0.49–4.66); gradient experiment: 42.6 (10–74) and 0.14 (0.03–0.30), respectively (see Supplementary Table S3 for values on individual treatments). In the crossing experiment, a 3 °C increase in temperature had a slight positive effect on the biomass of *Microcystis* and on the level of hepatotoxins (microcystins) (T, Fig. 1a,b), whereas brownification alone did not (B, Fig. 1a,b). However, the temperature effects on *Microcystis* and toxin concentrations were strongly amplified when combined with brownification (TB, Fig. 1a,b; Supplementary Table S2). Furthermore, in the gradient experiment, the *Microcystis* biomass and toxin levels increased considerably at only moderate changes in temperature and brownification (2 °C elevated temperature and 100% elevated absorbance, Fig. 1c,d; Supplementary Table S2), and reached a maximum effect size at the 3 °C and 150% increase in brownification. Although extrapolating findings from mesocosm studies to natural systems should be done with caution^24^, these results suggest that even those climate mitigation strategies that are aimed at achieving the 2 °C target^12^ will not be sufficient in preventing the degradation of freshwater quality.

The increase in microcystin concentrations in both experiments was not only due to elevated biomasses of *Microcystis*, but the ratio of microcystin to *Microcystis* biomass also increased (Supplementary Table S2), suggesting that either each cell produced more toxins or that the synergic interaction between brownification and temperature selected for more toxic strains. Although it is beyond the scope of this study to assess those mechanisms, our results show strong support for increased levels of cyanobacterial toxins in future waters. Our screening analyses for toxins (i.e., using an enzyme-linked immunosorbent assay; ELISA)^25^ provided information regarding the potential effects of warming and brownification on the total bulk of microcystins. However, we encourage further research on potential changes among different microcystin congeners, since temperature changes may alter the cellular synthesis of different microcystins in different ways^26^, and those variants may differ considerably in their toxicity^27^.

**Figure 2:**
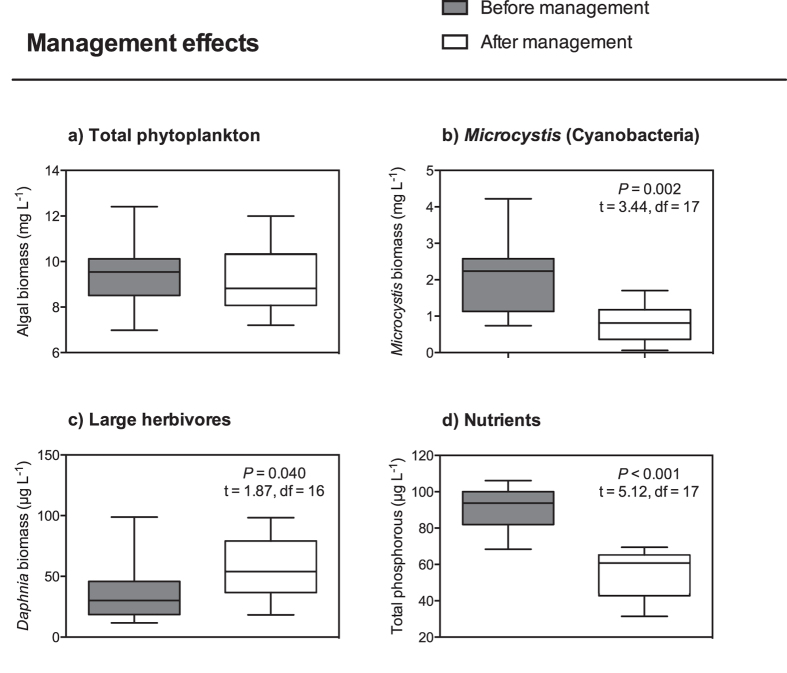
The effects of management through biomanipulation in Lake Ringsjön. Pairwise comparisons between the periods before (1996–2004; grey boxes) and after (2005–2014; white boxes) biomanipulation in the lake, which started in 2005. Boxplots show the minimum, first quartile, median, third quartile and maximum values of the monthly means from April to October from each year of (**a**) total phytoplankton biomass (mg L^−1^), (**b**) *Microcystis* biomass (mg L^−1^), (**c**) *Daphnia* biomass (μg L^−1^) and (**d**) total phosphorous concentrations (μg L^−1^). *P*-values, t-values and degrees of freedom are provided for significant differences (α = 0.05) based on a Student’s t-test (unpaired, one-tailed).

**Figure 3:**
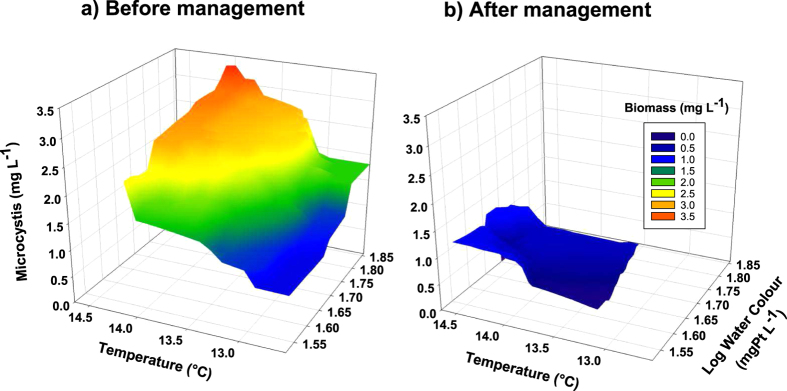
The effects of climatic warming and brownification on *Microcystis* in Lake Ringsjön. The response of *Microcystis* biomass (mg L^−1^) to temperature (°C) and water colour (used as a proxy of brownification; mg Pt L^−1^) (a) before management (1996–2004; n = 9) and (b) after management (2005–2014; n = 10) through biomanipulation. The surface 3D plots were produced through interpolation of the raw data (monthly means April–October from each year) using inverse square weighting. The multiple linear regression model for the period preceding biomanipulation (**a**) is: Y = −20.08 + 1.38*Temp + 2.21*Log Water Colour; F_*2,6*_ = 5.95, *P* = 0.019, R^2^ = 0.66, R^2^adj = 0.55. (**b**) There was no significant model that could be selected after biomanipulation was performed in the lake, i.e. *Microcystis* biomass was not related to variations in neither temperature nor water colour.

To explore whether the apparent synergistic effects of climate warming and brownification may be counteracted through local actions, we evaluated cyanobacterial levels before and after biomanipulation in Lake Ringsjön. While the total phytoplankton biomass did not change (Fig. 2a), the biomass of the dominant taxon, *Microcystis* (constituting up to 63% of the cyanobacterial biomass), was significantly higher before biomanipulation (Fig. 2b), despite the higher seasonal temperatures (+0.5 °C on average) during the period after biomanipulation (Supplementary Fig. S2). In the years before the adaptive management effort, the combined effect of brownification and temperature on *Microcystis* biomass was strong and varied by six-fold among years (Fig. 3a). Hence, results from a natural ecosystem are consistent with our experimental studies, which mechanistically tested the individual effects from temperature and brownification. Strikingly however, the same analysis performed on data collected from the years after the biomanipulation revealed no effects of temperature and brownification on *Microcystis* biomass development (Fig. 3b), suggesting that the local food web management buffered against these environmental perturbations. A likely cause for the disconnection of the changes in *Microcystis* biomass from the effects of temperature and humic substances in Lake Ringsjön after the biomanipulation was the higher biomass (nearly 50% increase) of large herbivorous zooplankton (*Daphnia*; Fig. 2c). This suggests that food web composition affects the responses of freshwater ecosystems to the combined pressures of climate warming and brownification^15^,^16^. Although the susceptibility of *Microcystis* to grazing by zooplankton is generally considered low, recent studies have noted relatively high grazing rates^28^,^29^,^30^. Furthermore, total phosphorous concentrations were almost 40% lower after the biomanipulation (Fig. 2d), and it should be noted that the phosphorus concentration dropped within a year after initiation of the biomanipulation efforts, following almost 10 years of steadily increasing concentrations (Supplementary Fig. S2). The main cause for the decrease in phosphorus was likely the removal of benthivorous fish, reducing its resuspension from the sediments into the water column^1^,^23^.

Average global temperatures have been projected to increase between 0.4 and 4.8 °C for the 21st century^12^. This is particularly serious for freshwater lakes, which are currently warming at unprecedented rates all around the globe^31^. This will intensify the reduction of clean water supplies in many regions, as well as increase metabolic rates and the water demands for energy and food production^12^. In addition, our results predict further degradation of eutrophic temperate freshwaters through increased levels of toxic cyanobacteria as the elevation of temperature in combination with humic run-off proceeds during the coming decades. However, our results also identify possibilities to locally counteract those trends in these affected systems and thereby improve water management planning against negative effects of global environmental changes^6^.

Efforts to secure our water resources, their biodiversity and their ecosystem resilience against global environmental change are emerging as mainstays in adaptation schemes for local actions and communities^32^,^33^. Besides the necessity for global action (e.g., the United Nations and the Ramsar conventions) in the development of effective global change adaptation strategies, water security implementation should be tailored to local conditions and authorities^32^,^33^. This is especially true considering that different regions on Earth will differ greatly in sensitivity to global environmental change^12^ and that the local and regional environment could influence the implementation of adaptation schemes. For example, despite the ability of cyanobacteria to tolerate low light intensities^18^, brownification can strongly supress primary production due to light limitation after surpassing a critical threshold^8^,^21^. Therefore, the amplifying effects of brownification in combination with warming on cyanobacterial growth are likely to occur in eutrophic systems with low-to-intermediate inputs of organic carbon^8^,^34^; consequently, this is likely to occur in watersheds with both forested land cover and high nutrient levels as a result of agriculture and/or urbanisation, such as in our study lake (Lake Ringsjön)^35^. Nevertheless, a reduction of water quality in less humic systems due to high levels of cyanobacterial toxins can dramatically compromise the supply of available clean water resources for many local communities, especially because humic lakes are not suitable for drinking purposes, given the high costs of removing humic substances. Furthermore, although biomanipulation has proven efficient in many systems^22^,^36^, the outcome is influenced by a lake´s trophic status^37^ and the likelihood for success seems to increase in geographical regions at higher latitudes. For example, fish diversity, omnivory and densities are generally higher in the tropics compared to temperate systems, which increase top-down control on zooplankton^38^. In addition, fish reproduction occurs throughout the year in warmer climates, which hinders the effectiveness of biomanipulations in controlling the recruitment of young-of-the-year fish^38^. Therefore, in contrast to temperate lakes, the potential to use biomanipulation to prevent the negative effects of global environmental change in tropical regions will greatly depend on our capacity to improve current methods for controlling fish predation.

In conclusion, our study identifies a strong empirical link between predicted changes in climate warming and humic content and the incidence of toxic cyanobacterial blooms, which are worldwide threats to the quality of future water resources, as well as to the biodiversity and ecosystem function of freshwater systems^3^,^4^,^5^. Strong interactions among environmental factors, such as temperature, humic substances and nutrients, are considered crucial for bloom formation by various cyanobacterial taxa, including the widespread and toxic *Microcystis*^39^,^40^,^41^. Counteracting climate warming and its potential interactions with other environmental stressors will take time to implement at the global scale, both with respect to political decision-making and executing solutions for mitigation. In the meantime, adaptive management are urgently needed to secure ecosystem function and the quality of natural resources^13^,^14^. We here provide strong empirical evidence that food web management is a promising tool for improving ecosystem resilience against harmful cyanobacteria. To our knowledge, no previous study has evaluated the interactive effects of biomanipulations with global environmental changes – neither with warming nor brownification. The integration of such local-scale actions into climate change adaptation schemes has the potential to provide a temporal buffer until global-scale solutions to mitigate greenhouse gas emissions are effectively established.

## Material and Methods

### Mesocosm experiments

#### Set-up and maintenance

Two long-term mesocosm experiments, ‘the crossing experiment’ and ‘the gradient experiment’, were run April–September 2011 and April–October 2013, respectively. The experiments consisted of 24 and 20 insulated cylindrical polyethylene enclosures (diameter: 0.7 m; height: 1 m; volume: 400 L), respectively, which were placed outdoors (open to the atmosphere) and on the ground at Lund University (55° 42′ N 13° 12′ E). The crossing experiment consisted of four experimental treatments (n = 6, randomly assigned to different treatments), including: ambient environmental conditions (C), warming of 3 °C above the ambient temperature (T), a doubling of humic substances above the ambient conditions (B), and a representation of the future environmental conditions, with the increases in temperature and humic substances (TB) combined. In the gradient experiment, five treatments (n = 4) aimed at a stepwise elevation both in temperature and brownification from ambient conditions (C-treatment), to TB1, +1 °C and +50% humic substances; TB2, +2 °C and +100%; TB3, +3 °C and +150%; and, finally, TB4, +4 °C and +200% humic substances. These climate conditions followed temperature projections from the IPCC^12^ and historical brownification trends of southern Swedish lakes^16^. Our studies focus on shallow lake ecosystems, which are common systems in urban and agricultural areas, and do not take stratification processes that occur in deep lakes into account.

To initiate the experiments, we placed lake sediment collected from Lake Krankesjön (55° 42′ N, 13° 27′ E) at the bottom of the mesocosms after a previous homogenisation of all sediment collected. The sediment was collected at a 1m-depth by using hand nets and transported in dark plastic boxes to the experimental facilities within two hours of sampling. We then covered the sediment with 400 L of unfiltered lake water. Lake Krankesjön is a shallow eutrophic lake (mean depth: 1.5 m) in a catchment dominated by agriculture with some forest and has relatively low water colour and total organic carbon concentrations compared to humic lakes (Supplementary Table S1). Two juvenile fish of the planktivorous species *Perca fluviatilis* and *Carassius carassius* (approximate length and weight; 50 mm and 5 g), which are common in shallow eutrophic lakes, were added to each enclosure in order to maintain high fish predation on zooplankton. The temperature was controlled by a computerized system, which instantaneously regulated the temperature from the heated enclosures in relation to that in the controls using real-time temperature sensors and heaters (Jäger 150 W). Hence, the diurnal and seasonal ambient temperature changes in the control treatments were mirrored at the specified elevated temperature levels in the heated treatments^16^. To avoid temperature differences in the mesocosms, a gentle airflow was induced inside a small Plexiglas tube in which the heater was mounted at one side of each mesocosm. To increase the humic substances in the crossing experiment brownification treatments, we used 80 L of 20 μm filtered lake water from Lake Liasjön (56° 26′ N, 13° 59′ E), which is a humic lake in a forested catchment with high water colour and total organic carbon concentrations (Supplementary Table S1). Considering the strength of the relationship between dissolved organic carbon concentrations and water colour based on absorbance measurements at 420 nm^42^, our experimental treatments were created by using water colour as a proxy for brownification. As a result, water additions from Lake Liasjön into our brownfication treatments (B and TB) resulted, on average, in an 81% increase in absorbance at 420 nm compared to control conditions. As a stronger increase in absorbance was needed in the brownest treatments in the gradient experiment, we here used commercially available humic and fulvic acids (HuminFeed®, Humintech, Germany), constituting the main fractions of natural humic matter in soils and freshwaters. An analysis of the relationship between the amount of commercial humic substances and absorbance at 420 nm was used to create a stock solution in distilled water (1 g L^−1^), from which a specific volume was added to each treatment, ranging from 0.4 L (TB1) to 1.5 L (TB4). Although the aim was to create a gradient from 0 to 200% increase (see above), the realized gradient reached 35%, 79%, 102% and 136% increases in absorbance in the TB1 to TB4 treatments, respectively, compared to the C treatment. The absorbance increase was maintained weekly by adding either 20 μm filtered humic water from Lake Liasjön (crossing experiment) or humic substance solution (gradient experiment), based on weekly measurements of the absorbance at 420 nm. In addition, distilled water was added weekly to compensate for evaporation losses, and the walls of the containers were scrubbed to minimize the growth of periphytic algae. The algae that were scrubbed off were allowed to sink and settle in order to recycle the nutrients and thereby keep the total nutrient levels similar in all enclosures. In order to avoid nutrient limitation in the enclosures, 1 mL of commercially available plant nutrients (Blomstra växtnäring, Cederroth, Upplands Visby, Sweden) was added bi-weekly to the enclosures in all treatments in both experiments.

#### Sample collection and analysis

The enclosures were sampled every second week from the surface to 0.1 m above the bottom using a Plexiglas tube (length: 1 m; diameter: 70 mm). Three samples were taken across the diameter of each mesocosm and were pooled (10 L). Subsamples were then taken for phytoplankton counts, chlorophyll-*a*, cyanotoxins (microcystins), water colour and nutrient analyses. The phytoplankton subsamples were immediately preserved in Lugol’s solution and stored at 4 °C. Cyanobacteria were counted and determined to genus level by using tubular chambers and an inverted microscope (Olympus IX53)^40^. The total chlorophyll-*a* concentrations were measured with a fluorometer (AlgaeLabAnalyser, ALA, bbe moldaenke, Germany) within an hour of sampling. The microcystin subsamples were immediately stored at −20 °C after sampling and analysed as equivalents of the variant microcystin-LR, using an enzyme-linked immunosorbent assay (ELISA)^40^. The water colour was measured as absorbance at 420 nm on a spectrophotometer (Beckman DU800 Coulter), after filtration through a glass microfiber filter (Grade GF/C, Whatman^TF^). Finally, total phosphorus was analysed as soluble reactive phosphorus after digestion with potassium persulphate.

#### Data analyses

Two-Way Repeated Measures Analysis of Variance (RM-ANOVA), with time and the warming and brownification treatments as factors, was used to analyse the treatment effects throughout the experiment on the total phosphorous concentrations, total phytoplankton biomass, *Microcystis* biomass and microcystin concentrations. Dunnett´s multiple comparison *post hoc* was used to identify significant differences among treatments. We also used mean temporal effect sizes (±SE) calculated from each sampling occasion to represent the standardised treatment responses across the crossing and gradient experiments for the growth of *Microcystis* and the concentrations of their hepatotoxins (microcystins)^43^. Statistical analyses were performed with SPSS 21 for Macintosh and plots were created with GraphPad Prism.

### Field study

Lake Ringsjön (55° 53′ N, 13° 28′ E) is a shallow eutrophic lake with a surface area of 40 km^2^. The lake is located in a catchment consisting of 40% forests and arable land each^35^ and has low total organic carbon concentrations and water colour compared to humic lakes (Supplementary Table S1). The lake is used as a drinking water reservoir and for recreational purposes. Since 1980, Lake Ringsjön has experienced climatic warming of around 0.8 °C in mean water temperature and a doubling in the concentrations of humic substances^16^. The lake became eutrophic in the 1970s and a program to control the level of nutrients was introduced in the 1980s, which reduced the external loading of phosphorous from 30 tonnes to 5–10 tonnes per year. To further improve the water quality, a ‘biomanipulation’ program was started in 2005, for which both planktivorous (roach, *Rutilus rutilus*, and small perch, *Perca fluviatilis*) and benthivorous fish (bream, *Abramis brama*), were removed (Supplementary Fig. S1).

#### Sample collection and analysis

Daily measurements of air temperature taken by the Swedish Meteorological and Hydrological Institute (SHMI) meteorological station (55° 69′ N, 13° 22′ E) were used as a proxy for climatic warming in Lake Ringsjön. The lake consists of three connected basins; in the western basin (mean depth: 3.1 m; maximum depth: 5.4 m), the water colour (humic substances), total phosphorous, and the phytoplankton and zooplankton community structure, have been monitored since 1996. Samples were taken monthly from April to October each year at the location of maximum water depth in the lake. An integrated sample (10–30 L) of the water column was taken with a Plexiglas sampler from which subsamples for phytoplankton, total phosphorous and water colour analyses were collected. The remaining water was filtered through a 50-μm mesh to collect the zooplankton, which we stored in 100 mL bottles. Phytoplankton and zooplankton samples were immediately preserved in Lugol’s solution and stored at 4 °C after sampling. Phytoplankton (including cyanobacteria) were counted to genus level and their biomass determined using tubular chambers and an inverted microscope (Olympus IX 53), according to the methods detailed in the mesocosm experiments (above). Large zooplankton (*Daphnia* spp.) were counted to genus level using a stereoscopic microscope (Olympus SZX7) at ×20 magnification and their biomass was estimated using length–weight regressions^29^. Because monitoring of dissolved organic carbon concentrations or water color based on absorbance measurements (abs 420 nm) were not available for the entire study period (1996–2014), we used water colour measurements by using a standard PtCl_6_^−2^ solution^44^. Total phosphorus was analysed as soluble reactive phosphorus after digestion with potassium persulphate.

#### Data analyses

Student’s t-tests were used to explore the effects of biomanipulation on the phytoplankton community structure, that is, on the biomasses of total phytoplankton and *Microcystis*. This was done by comparing the years before (1996–2004; n = 9) and after (2005–2014, n = 10) the biomanipulation, using seasonal means that were derived from the monthly values (April–October). In addition, we compared the effects of the biomanipulation through Student’s t-tests (before and after) on potentially explanatory variables that may have affected cyanobacterial growth, that is, *Daphnia* spp. (large-bodied herbivores) biomass and total phosphorous concentrations. Next, we used multiple linear regression analyses to explore the relationships between the biomasses of *Microcystis* (as dependent variable) and the temperature and brownification (as predictor variables), for the periods before and after the biomanipulation (separately). Further, we produced surface 3D plots for the two periods to represent the development of the *Microcystis* biomass as a function of the temperature and water colour. All variables were log transformed before statistical analysis, except for the *Microcystis* biomass and temperature. Statistical analyses were performed with SPSS 21 for Macintosh.

## Additional Information

**How to cite this article**: Urrutia-Cordero, P. *et al*. Local food web management increases resilience and buffers against global change effects on freshwaters. *Sci. Rep.* **6**, 29542; doi: 10.1038/srep29542 (2016).

## Supplementary Material

Supplementary Information

## References

[b1] Scheffer M. Ecology of Shallow Lakes. (Chapman and Hall, 1998).

[b2] Schindler D. W. Recent advances in the understanding and management of eutrophication. Limnol Oceanogr 51, 356–363 (2006).

[b3] Codd G. A., Morrison L. F. & Metcalf J. S. Cyanobacterial toxins: risk management for health protection. Toxicol Appl Pharm 203, 264–272 (2005). 10.1016/j.taap.2004.02.016 15737680

[b4] Paerl H. W. & Huisman J. Climate - Blooms like it hot. Science 320, 57–58 (2008). 18388279 10.1126/science.1155398

[b5] Brookes J. D. & Carey C. C. Resilience to blooms. Science 333, 46–47 (2011). 21980099 10.1126/science.1207349

[b6] Reid W. . Millennium Ecosystem Assessment: Ecosystem and human well-being: synthesis. (Washington, 2005).

[b7] Monteith D. T. . Dissolved organic carbon trends resulting from changes in atmospheric deposition chemistry. Nature 450, 537–U539 (2007). 18033294 10.1038/nature06316

[b8] Solomon C. T. . Ecosystem consequences of changing inputs of terrestrial dissolved organic matter to lakes: current knowledge and future challenges. Ecosystems 18, 376–389 (2015).

[b9] Granéli W. Brownification of lakes. In *Encyclopedia of* Lakes *and Reservoirs* (ed L. Bengtsson, Herschy, R. W., Fairbridge, R.) 117–119 (Springer, 2012).

[b10] Kritzberg E. S. & Ekström S. M. Increasing iron concentrations in surface waters - a factor behind brownification? Biogeosciences 9, 1465–1478 (2012).

[b11] Weyhenmeyer G. A., Muller R. A., Norman M. & Tranvik L. J. Sensitivity of freshwaters to browning in response to future climate change. Climatic Change 134, 225–239 (2016).

[b12] Stocker, T. F. & al., e. The physical science basis, IPCC 2013. (Cambridge University Press, Cambridge, 2013).

[b13] Pace M. L., Carpenter S. R. & Cole J. J. With and without warning: managing ecosystems in a changing world. Front Ecol Environ 13, 460–467 (2015).

[b14] Scheffer M. . Creating a safe operating space for iconic ecosystems. Science 347, 1317–1319 (2015). 25792318 10.1126/science.aaa3769

[b15] Alsterberg C., Eklöf J. S., Gamfeldt L., Havenhand J. N. & Sundbäck K. Consumers mediate the effects of experimental ocean acidification and warming on primary producers. P Natl Acad Sci USA 110, 8603–8608 (2013). 10.1073/pnas.1303797110 PMC3666745 23630263

[b16] Hansson L.-A. . Food-chain length alters community responses to global change in aquatic systems. Nature Climate Change 3, 228–233, 10.1038/nclimate1689 (2013).

[b17] Robarts R. D. & Zohary T. Temperature effects on photosynthetic capacity, respiration, and growth-rates of bloom-forming cyanobacteria. New Zeal J Mar Fresh 21, 391–399 (1987).

[b18] Carey C. C., Ibelings B. W., Hoffmann E. P., Hamilton D. P. & Brookes J. D. Eco-physiological adaptations that favour freshwater cyanobacteria in a changing climate. Water Res 46, 1394–1407 (2012). 22217430 10.1016/j.watres.2011.12.016

[b19] Paerl H. W. & Huisman J. Climate change: a catalyst for global expansion of harmful cyanobacterial blooms. Env Microbiol Rep 1, 27–37 (2009). 23765717 10.1111/j.1758-2229.2008.00004.x

[b20] Williamson C. . Ecological consequences of long-term browning in lakes. Scientific reports 5, 18666, 10.1038/srep18666 (2015). PMC4687041 26690504

[b21] Seekell D. A. . The influence of dissolved organic carbon on primary production in northern lakes. Limnol Oceanogr 60, 1276–1285 (2015).

[b22] Hansson L.-A. . Biomanipulation as an application of food chain theory: constraints, synthesis and recommendations for temperate lakes. Ecosystems 1, 558–574 (1998).

[b23] Bergman E. . Synthesis of the theoretical and empirical experiences from nutrient and cyprind reductions in Lake Ringsjön. Hydrobiologia 404, 145–156 (1999).

[b24] Stewart R. I. A. . Mesocosm experiments as a tool for ecological climate-change research. Advances in Ecological Research 48, 171–181 (2013).

[b25] Moreira C., Ramos V., Azevedo J. & Vasconcelos V. Methods to detect cyanobacteria and their toxins in the environment. Appl Microbiol Biot 98, 8073–8082 (2014). 10.1007/s00253-014-5951-9 25085613

[b26] Rapala J., Sivonen K., Lyra C. & Niemela S. I. Variation of microcystins, cyanobacterial hepatotoxins, in Anabaena spp. as a function of growth stimuli. Appl Environ Microb 63, 2206–2212 (1997). 10.1128/aem.63.6.2206-2212.1997 PMC168513 9172340

[b27] Gupta N., Pant S. C., Vijayaraghavan R. & Rao P. V. L. Comparative toxicity evaluation of cyanobacterial cyclic peptide toxin microcystin variants (LR, RR, YR) in mice. Toxicology 188, 285–296 (2003). 12767698 10.1016/s0300-483x(03)00112-4

[b28] Chislock M. F., Sarnelle O., Olsen B. K., Doster E. & Wilson A. E. Large effects of consumer offense on ecosystem structure and function. Ecology 94, 2375–2380 (2013). 24400489 10.1890/13-0320.1

[b29] Urrutia-Cordero P., Ekvall M. K. & Hansson L.-A. Responses of cyanobacteria to herbivorous zooplankton across predator regimes: who mows the bloom? Freshwater Biol 60, 960–972 (2015).

[b30] Urrutia-Cordero P., Ekvall M. K. & Hansson L.-A. Controlling harmful cyanobacteria: Taxa-specific responses of cyanobacteria to grazing by large-bodied *Daphnia* in a biomanipulation scenario. Plos One 10.10.1371/journal.pone.0153032 (2016). PMC4820120 27043823

[b31] O’Reilly C. M. . Rapid and highly variable warming of lake surface waters around the globe. Geophys Res Lett 42 (2015).

[b32] Hering J. G. . Local perspectives on water. Science 349, 479–480 (2015). 26228131 10.1126/science.aac5902

[b33] Vorosmarty C. J., Hoekstra A. Y., Bunn S. E., Conway D. & Gupta J. What scale for water governance? Science 349, 478–479 (2015). 26228130 10.1126/science.aac6009

[b34] Hanson P. C., Bade D. L. & Carpenter S. R. Lake metabolism: relationships with dissolved organic carbon and phosphorus. Limnol. Oceanogr. 48, 1112–1119 (2003).

[b35] Hansson L.-A. & Bergman E. Nutrient reduction and biomanipulation as tools to improve water quality: The Lake Ringsjön story. Vol. DH140 (Kluwer Academic Publishers, 1999).

[b36] Bernes C. . What is the influence of a reduction of planktivorous and benthivorous fish on water quality in temperate eutrophic lakes? A systematic review. Environmental Evdence 4 (2015).

[b37] Elser J. J. & Goldman C. R. Zooplankton effects on phytoplankton in lakes of contrasting trophic status. Limnol Oceanogr 36, 64–90 (1991).

[b38] Jeppesen E. . Restoration of shallow lakes by nutrient control and biomanipulation-the successful strategy varies with lake size and climate. Hydrobiologia 581, 269–285 (2007).

[b39] Kosten S. . Warmer climates boost cyanobacterial dominance in shallow lakes. Global Change Biol 18, 118–126 (2012).

[b40] Ekvall M. K. . Synergistic and species-specific effects of climate change and water colour on cyanobacterial toxicity and bloom formation. Freshwater Biol 58, 2414–2422 (2013).

[b41] Rigosi A., Carey C. C., Ibelings B. W. & Brookes J. D. The interaction between climate warming and eutrophication to promote cyanobacteria is dependent on trophic state and varies among taxa. Limnol Oceanogr 59, 99–114 (2014).

[b42] Pace M. L. & Cole J. J. Synchronous variation of dissolved organic carbon and color in lakes. Limnol Oceanogr 47, 333–342 (2002).

[b43] Cohen J. Statistical power analysis for the behavioural sciences. 2nd edn, (Hillsdale, NJ: Lawrence Erlbaum, 1988).

[b44] Wetzel R. G. & Likens G. Limnological analyses. (Springer, 2000).

