# Supplementary material for: Local food web management increases resilience and buffers against global change effects on freshwaters
Source: Sci Rep. 2016 Jul 8;6:29542. doi: 10.1038/srep29542 (PMC4937379; doi:10.1038/srep29542)
Supplement: Supplementary Information [file srep29542-s1.pdf]

SUPPLEMENTARY MATERIAL

Local food web management increases resilience and buffers against global change effects on freshwaters

Authors: Pablo Urrutia-Cordero[1,2,], Mattias K. Ekvall[1], and Lars-Anders Hansson[1*]

[1]Department of Biology/Aquatic Ecology, Lund University, Ecology building, SE-223 62 Lund, Sweden

[2]Center for Environmental and Climate Research, Lund University, Ecology Building, SE-223 62, Lund, Sweden

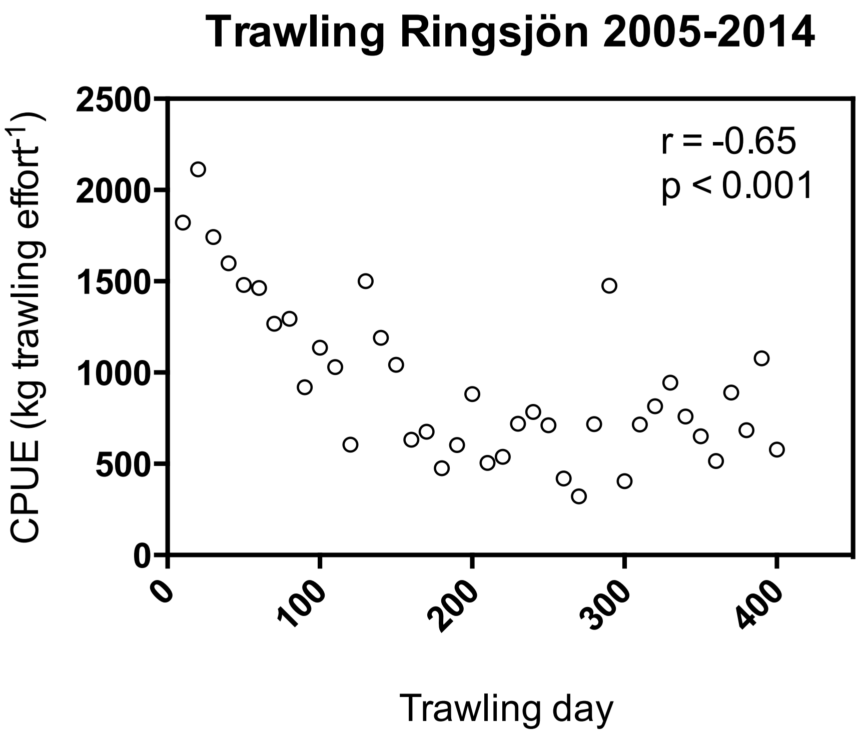

Supplementary Fig. S1: Biomanipulation (trawling) in Lake Ringsjön. The relationship between the cumulative number of trawling days (X-axis) and the catch per unit effort (CPUE; catch in kg per 10 trawling days; Y-axis) of roach, bream and small perch in Lake Ringsjön. The decrease in CPUE indicates a reduced number of fish in the lake. Note that the cumulative number of trawling days expands from the start (2005) to the end of the biomanipulation (2014).

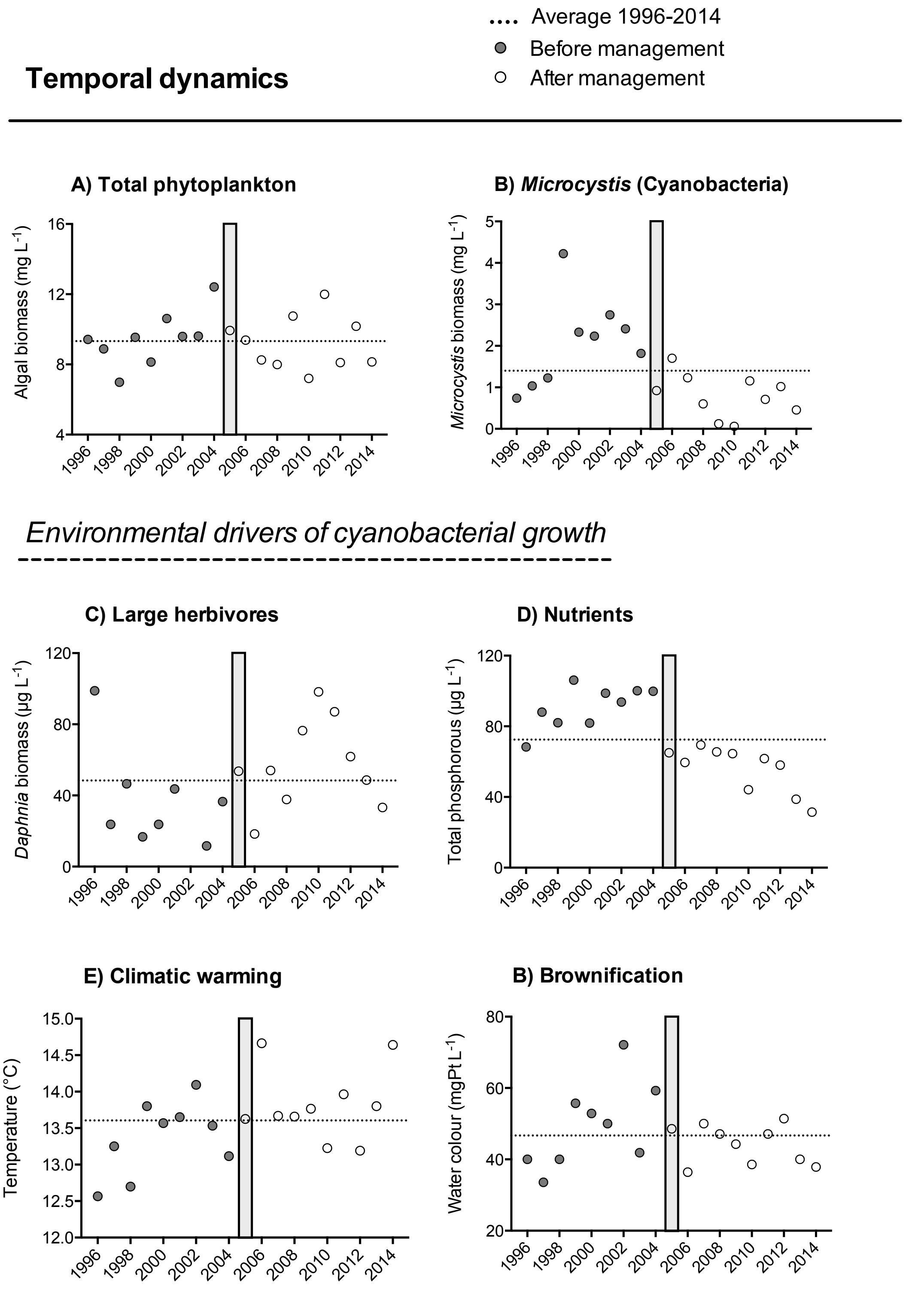
Average 1996-2014
Before management
After management
Temporal dynamics
A) Total phytoplankton
Algal biomass (mg L-1)
B) Microcystis (Cyanobacteria)
Microcystis biomass (mg L-1)
Environmental drivers of cyanobacterial growth
C) Large herbivores
Daphnia biomass (µg L-1)
D) Nutrients
Total phosphorous (µg L-1)
E) Climatic warming
Temperature (°C)
B) Brownification
Water colour (mgPt L-1)

Supplementary Fig. S2: **Biological, chemical and environmental data from Lake Ringsjön**. Temporal dynamics during the periods before management (1996–2004; grey circles) and after management (2005–2014; white circles) through biomanipulation in Lake Ringsjön of (A) total phytoplankton biomass (mg L^{-1}), (B) *Microcystis* biomass (mg L^{-1}), (C) *Daphnia* biomass (μg L^{-1}), (D) total phosphorous concentrations (μg L^{-1}), (E) temperature (°C) and (F) water color (used as a proxy of brownification; mg Pt L^{-1}). Values are seasonal means, derived from monthly (April–October) values measured during each year, i.e. each data point is based on seven samplings. The dashed line represents the average level during the whole the study period (1996–2014) and the grey bar denotes the start of biomanipulation in 2005.

CHEMICAL CHARACTERISTICS OF LAKES KRANKESJÖN, LIASJÖN AND RINGSJÖN

Supplementary Table S1: Monitoring data (total phosphorous, abs_{420} and total organic carbon) from our study systems, Lakes Krankesjon, Liasjön and Ringsjön. Data represent means (±SD) for August samples from 1998 to 2013 (source: national monitoring, http://webstar.vatten.slu.se/db.html).

**Lake**	**Tot-P (μg L^{-1})**	**Abs_{420} (cm^{-1})**	**TOC (mg L^{-1})**
Krankesjön	43.3 ± 15.8	0.055 ± 0.016	14.2 ± 3.7
Liasjön	35.1 ± 15.2	0.194 ± 0.049	28.5 ± 9.0
Ringsjön	112.1 ± 31.1	0.049 ± 0.013	11.4 ± 2.4

STATISTICS: MESOCOSM EXPERIMENTS

Supplementary Table S2: Two-Way Repeated Measures ANOVA with the warming/brownification treatments and time as factors for both the crossing and gradient experiments for total phosphorous concentrations (µg L^{-1}), total phytoplankton biomasses (chlorophyll *a*; µg L^{-1}), *Microcystis* biomasses (mg L^{-1}), microcystin concentrations (µg L^{-1}) and microcystin to *Microcystis* ratio. In the crossing experiment: T is elevated temperature (+3°C); B is brownification (double absorbance at 420 nm (used as a proxy for brownification)); and TB is the combination of both factors. In the gradient experiment: TB1 is +1°C and +50% absorbance; TB2 is +2°C and +100% absorbance; TB3 is +3°C and +150% absorbance; and TB4 is +4 °C and +200% absorbance. *Post hoc* analyses (Dunnett´s test) display significant ($P < 0.05$) differences between treatments and the control. NS = non-significant results.

**Crossing experiment**	**Total phosphorous**			**Total phytoplankton biomass**			***Microcystis* biomass**			**Microcystin concentrations**			**Microcystin to *Microcystis* ratio**		
	F	Dfn, DFd	*P*	*F*	Dfn, DFd	*P*	*F*	Dfn, DFd	*P*	*F*	Dfn, DFd	*P*	*F*	Dfn, DFd	*P*
Time	53.5	4, 80	**< 0.001**	42.2	8, 160	**< 0.001**	20.2	9, 180	**< 0.001**	35.8	8, 160	**< 0.001**	10.0	8, 160	**< 0.001**
Factor x Time	0.3	12, 80	NS	1.9	24, 160	**0.007**	1.3	27, 180	NS	1.4	24, 160	NS	2.1	24, 160	**0.003**
Factor	0.6	3, 20	NS	1.4	3, 20	NS	3.4	3, 20	**< 0.001**	6.9	3, 20	**0.002**	7.4	3, 20	**0.002**
Post hoc		–			–			C-TB			C-TB			C-TB	
**Gradient experiment**	**Total phosphorous**			**Total phytoplankton biomass**			***Microcystis* biomass**			**Microcystin concentrations**			**Microcystin to *Microcystis* ratio**		
	F	Dfn, DFd	*P*	*F*	Dfn, DFd	*P*	*F*	Dfn, DFd	*P*	*F*	Dfn, DFd	*P*	*F*	Dfn, DFd	*P*
Time	54.4	10, 150	**< 0.001**	71.3	14, 210	**< 0.001**	84.8	14, 210	**< 0.001**	47.4	14, 210	**< 0.001**	35.3	14, 210	**< 0.001**
Factor x Time	1.2	40, 150	NS	1.9	56, 210	**< 0.001**	3.4	56, 210	**< 0.001**	3.3	56, 210	**< 0.001**	4.3	56, 210	**< 0.001**
Factor	1.6	4, 15	NS	2.1	4, 15	NS	7.7	4, 15	**0.001**	9.0	4, 15	**< 0.001**	6.5	4, 15	**0.003**
Post hoc		–			–			C-TB2, C-TB3, C-TB4			C-TB2; C-TB3, C-TB4			C-TB3	

MICROCYSTIS BIOMASS AND MICROCYSTIN CONCENTRATIONS IN THE MESOCOSM EXPERIMENTS

Supplementary Table S3: *Microcystis* biomass (µg L^{-1}) and microcystin concentrations (ppb) in each individual treatment (treatment n=4, total n=20) for both the crossing and the gradient experiment. Mean seasonal values are calculated across all sampling occasions.

**Crossing experiment**	***Microcystis* biomass**		**Microcystin concentrations**	
	Mean	Range	Mean	Range
Control	329	34-823	1.10	0.20-2.18
T	1632	66-7401	1.43	0.12-2.94
B	214	38-562	0.49	0.07-0.84
TB	1332	112-3058	4.66	0.23-8.30
**Gradient experiment**	***Microcystis* biomass**		**Microcystin concentrations**	
	Mean	Range	Mean	Range
Control	10	0-24	0.03	0.00-0.06
TB1	10	0-30	0.03	0.00-0.08
TB2	53	0-125	0.16	0.00-0.51
TB3	66	2-163	0.30	0.08-0.86
TB4	74	4-179	0.20	0.02-0.59
